# Key role of thymic stromal lymphopoietin as a molecular target for biologic treatment of severe asthma

**DOI:** 10.3389/falgy.2025.1671353

**Published:** 2025-11-14

**Authors:** Corrado Pelaia, James Melhorn, Giovanni Paoletti, Remo Poto, Angelantonio Maglio, Claudia Crimi, Alessandro Vatrella, Giorgio Walter Canonica, Girolamo Pelaia

**Affiliations:** 1 Department of Medical and Surgical Sciences, University “Magna Graecia” of Catanzaro, Catanzaro, Italy; 2 Respiratory Medicine Unit, Nuffield Department of Medicine, University of Oxford, Oxford, United Kingdom; 3 Department of Biomedical Sciences, Humanitas University, Pieve Emanuele/Milano, Italy; 4 Personalized Medicine, Asthma and Allergy, Humanitas Clinical and Research Center, IRCCS, Rozzano/Milano, Italy; 5 Department of Translational Medical Sciences, University of Naples Federico II, Naples, Italy; 6 Center for Basic and Clinical Immunology Research (CISI), University of Naples Federico II, Naples, Italy; 7 Department of Medicine, Surgery, and Dentistry, University of Salerno, Salerno, Italy; 8 Department of Clinical and Experimental Medicine, University of Catania, Catania, Italy; 9 Department of Health Sciences, University “Magna Graecia” of Catanzaro, Catanzaro, Italy

**Keywords:** severe asthma endotypes, thymic stromal lymphopoietin, alarmins, airway epithelium, innate and adaptive immune responses, tezepelumab

## Abstract

Thymic stromal lymphopoietin (TSLP) is an alarmin mainly released by airway epithelial cells injured by many environmental noxious agents such as aeroallergens, respiratory viruses, bacteria, airborne pollutants and cigarette smoking. Airway expression levels of TSLP are related to both asthma severity and the extent of bronchial obstruction occurring in asthmatic patients. The pivotal pathogenic role played by TSLP in asthma is due to its capability of acting as an upstream driver of multiple cellular and molecular proinflammatory pathways, responsible for the development and persistence of both type 2 (T2-high) and T2-low asthma. Tezepelumab is a fully human monoclonal antibody which specifically binds to TSLP, thus impeding its interaction with the TSLP receptor complex expressed by immune/inflammatory and resident cells of the airways. By virtue of this very effective mechanism of action, tezepelumab prevents disease exacerbations and improves lung function. These positive outcomes have been verified by randomized clinical trials, as well as by preliminary real-life studies. The aim of this narrative review is to provide an overview of the pathogenic involvement of TSLP in asthma, followed by an updated discussion focused on the therapeutic effects induced by tezepelumab in severe asthmatic patients.

## 1 Introduction

The onset and persistence of asthma arise from complex interactions between genetic and environmental factors (1). This widespread respiratory disease is characterized by chronic bronchial inflammation associated with hyperresponsiveness and structural remodeling of the airways, that are all together responsible for chest tightness, wheezing, shortness of breath, cough and worsening of lung function (2, 3). Among more than 300 million asthmatic patients scattered around the world, about 5%–10% suffer from severe asthma, featured by the failure of even high doses of standard inhaled treatments to provide an adequate therapeutic control (4, 5). In particular, recurrent exacerbations are the main clinical expressions of severe asthma (6). Intricate networks of cellular, molecular, and immunopathologic mechanisms (endotypes) lead to the manifestation of several heterogeneous asthmatic traits (phenotypes) (7, 8). The latter include either allergic or non-allergic eosinophilic inflammatory profiles, as well as neutrophilic, mixed eosinophilic/neutrophilic, and paucigranulocytic patterns (9). All the above asthmatic endotypes/phenotypes are driven, maintained and amplified by an intense biosynthetic activity of the bronchial epithelium, triggered by tissue damage induced by several noxious agents such as allergens, viruses, bacteria, fungi, cigarette smoking and airborne pollutants (10, 11). In asthmatic subjects, basal airway epithelial cells are the main source of innate cytokines known as alarmins, including interleukin-25 (IL-25), interleukin-33 (IL-33), TNF-like cytokine 1A (TL1A), high-mobility group box 1 protein (HMGB1), and especially thymic stromal lymphopoietin (TSLP) (12–15).

**Figure 1:**
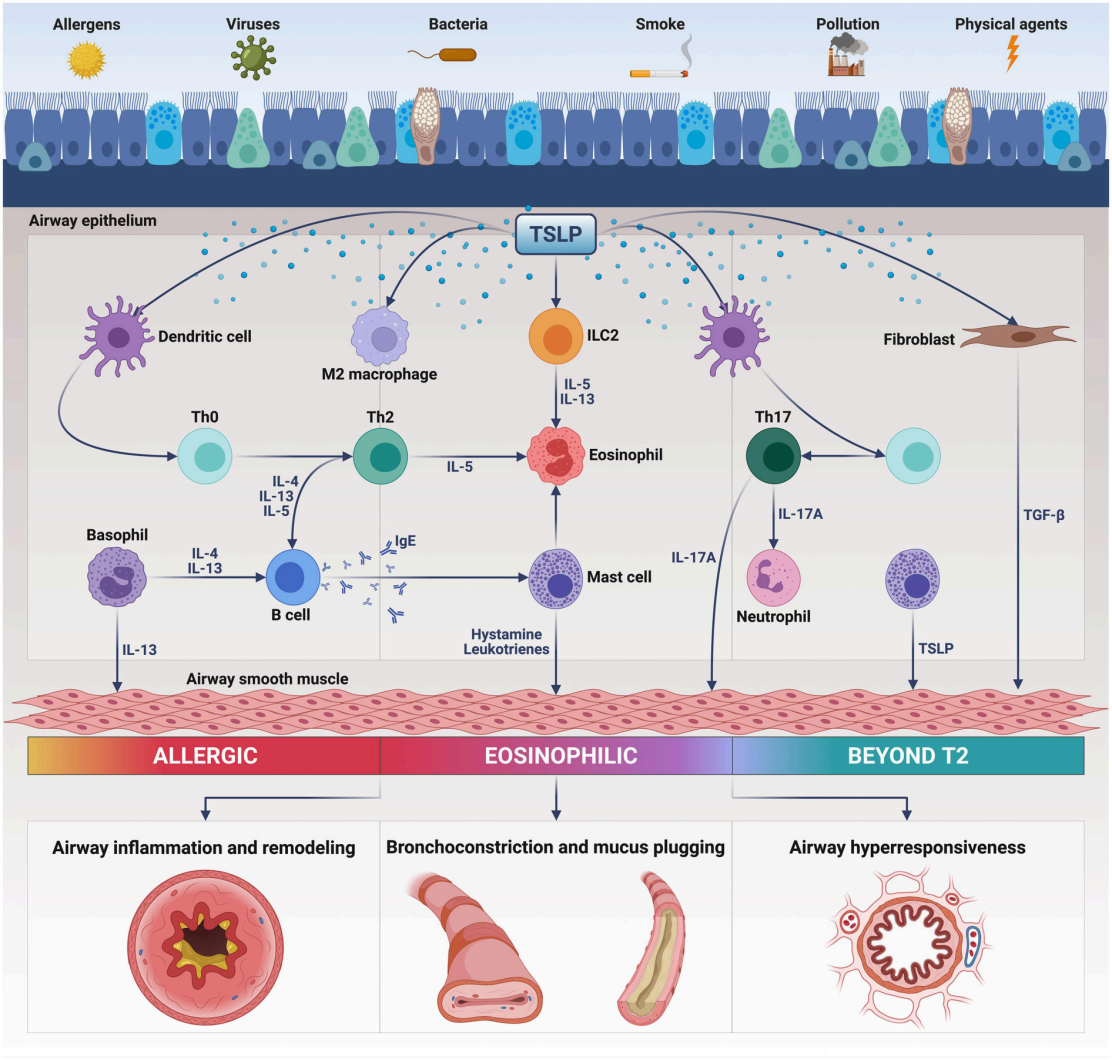
Pathogenic actions of thymic stromal lymphopoietin (TSLP) in asthma. Released from injured bronchial epithelium, TSLP triggers the inflammatory responses underlying T2-high and T2-low asthma by activating several immune cells including dendritic cells, Th2/Th17 lymphocytes, group 2 innate lymphoid cells (ILC2), eosinophils, mast cells, basophils, and M2 macrophages. TSLP also activates airway structural cells such as fibroblasts and smooth muscle cells. As a consequence of these pleiotropic actions, TSLP induces airway inflammation and remodeling, bronchial hyperresponsiveness, bronchoconstriction and mucus plugging. This original figure was created by the authors using “BioRender.com”.

Notably, TSLP is an alarmin that plays a pivotal pathobiologic role in T2-high eosinophilic asthma, as well as in T2-low neutrophilic disease (16) (Figure 1). Indeed, TSLP promotes the production of type 2 cytokines such as interleukins 4 (IL-4), 5 (IL-5) and 13 (IL-13), involved in eosinophilic inflammation, and also stimulates the immune/inflammatory responses mediated by interleukins 1 (IL-1) and 17 (IL-17), thereby eliciting airway neutrophilic infiltration (11, 16). TSLP thus exerts potent, either direct or indirect pleiotropic effects on T and B lymphocytes, group 2 innate lymphoid cells (ILC2), mast cells, basophils, macrophages, eosinophils and neutrophils (11, 16). Furthermore, TSLP contributes to bronchoconstriction and airway structural remodeling by activating the functions of resident cells like fibroblasts and smooth muscle cells (17, 18).

Because of its relevant pathogenic features, TSLP is a very suitable molecular target for biologic treatment of severe asthma (19, 20). In this regard, the fully human anti-TSLP monoclonal antibody tezepelumab has been developed and approved as anti-asthma biologic therapy (21–23). TSLP is effectively bound and sequestered by tezepelumab, which can be usefully utilized across T2-high and T2-low asthma phenotypes/endotypes (23, 24). In fact, both randomized clinical trials (RCTs) and real-life studies have proven that tezepelumab exerts a positive therapeutic impact on both eosinophilic and non-eosinophilic severe asthma (25–35).

On the basis of the above considerations, this narrative review aims to highlight the pathogenic role of TSLP in asthma, and also to provide an updated coverage of the therapeutic use of tezepelumab as add-on biologic therapy of severe asthma.

## 2 Basic mechanisms of the pathogenic role of TSLP in asthma

Initially identified as a B-lymphocyte trophic factor in thymic stromal cells, TSLP belongs to the interleukin-2 (IL-2) cytokine family, and its gene originated from early chromosomal duplication of the interleukin-7 (IL-7) gene (17, 36). Notably, in severe asthmatic patients a suggestive association was found with the rs1837253 single nucleotide polymorphism present in the TSLP gene, located on chromosome 5 (37). TSLP exists as a short, non-inducible isoform (60 amino acids) characterized by homeostatic/anti-inflammatory properties, and as a longer variant (159 amino acids) whose expression can be induced by proinflammatory stimuli (38–40). The positive charges of long TSLP interact with the negative charges of a heterodimer consisting of the TSLP receptor (TSLPR) and the α subunit of IL-7 receptor (IL-7Rα/CD127) (40, 41). Specifically, TSLP binding to TSLPR induces the recruitment of IL-7Rα and the consequent assembly of the ternary complex TSLP/TSLPR/IL-7Rα (11). The latter drives the engagement of an intricate signal transduction network including several kinases and transcription factors such as Janus kinases 1 and 2 (JAK1/2), mitogen-activated protein kinases (MAPKs), phosphatidylinositol 3-kinase (PI3 K), signal transducers and activators of transcription 3 and 5 (STAT3/5), and nuclear factor κB (NF-κB) (41–43). In particular, TSLP triggers the activation of JAK2 pathway via TSLPR, and stimulates the functions of JAK1 signaling module through IL-7Rα (17, 44).

High TSLP levels can be detected in epithelial bronchial biopsies taken from asthmatic subjects, as well as in their bronchoalveolar lavage fluid (BALF), exhaled breath condensate, induced sputum and serum (16, 45–47). Airway TSLP expression correlates with asthma severity and bronchial obstruction (16). It is also noteworthy that genome-wide association studies have highlighted that some single-nucleotide polymorphisms (SNPs), located inside the TSLP gene, outline variable degrees of susceptibility to asthma (48–50). Moreover, TSLP mRNA is overexpressed in nasal polyps excised from patients with aspirin-exacerbated respiratory disease (AERD) (51).

In asthmatic airways, bronchial epithelial cells are the main sources of TSLP, which is also produced by mast cells, basophils, eosinophils, dendritic cells, T lymphocytes, fibroblasts and airway smooth muscle cells (17, 52, 53). TSLP secretion can be elicited by a wide array of stimuli encompassing proinflammatory cytokines such as IL-4, IL-13, IL-25, IL-1β and TNF-α, as well as immunoglobulins E (IgE), protease containing aeroallergens, respiratory viruses, airborne pollutants and cigarette smoking (53, 54). After being released from bronchial epithelial cells, TSLP exerts its biologic effects on many cellular targets expressing the TSLPR/IL-7Rα heterodimer.

### 2.1 T2-high asthma

Namely, TSLP promotes the survival of ILC2 cells and stimulates their biosynthetic activity leading to the production of IL-4, IL-5, IL-9 and IL-13 (16, 55–57). IL-4 is essential for Th2 lymphocyte differentiation and IgE isotype switching, IL-5 is the pivotal eosinophilopoietic cytokine, IL-9 is a key mast cell growth factor, whereas IL-13 is responsible for goblet cell metaplasia, and also for bronchoconstriction, bronchial hyperresponsiveness and proliferation of airway smooth muscle cells (55, 58, 59). TSLP immunoreactivity and ILC2 share the same localization in the airways, and a close relationship has been found between TSLP amount and ILC2 counts in nasal biopsies obtained from subjects with severe asthma and chronic rhinosinusitis (CRS) (60, 61). Furthermore, TSLP causes ILC2 resistance to corticosteroids (62). Indeed, ex vivo studies have shown that dexamethasone is unable to inhibit cytokine production in ILC2 obtained from asthmatic patients characterized by the presence of high airway levels of TSLP (62).

Eosinophils and their progenitors also express the TSLPR/IL17-Rα receptor complex, and these cells quickly respond to TSLP that inhibits apoptosis thus lengthening cell survival, as well as elicits the release of eosinophilic pro-inflammatory chemokines and cytotoxic proteins (63, 64). All the pro-eosinophilic actions of TSLP are mediated by signal transduction pathways operated by MAPKs and NF-κB (63, 64). Interestingly, allergen challenge is followed by an overlapping localization of TSLP and eosinophilic infiltration within asthmatic airways (65). TSLP also enables eosinophils to assemble eosinophilic extracellular traps, made of complexes including cytotoxic proteins and mitochondrial DNA, which in asthmatic patients are implicated in innate immune responses and mucus hypersecretion (66). Moreover, TSLP acts on eosinophil progenitors by enhancing the expression of the α subunit of IL-5 receptor (IL-5Rα), thus contributing to stimulate IL-5-dependent maturation and differentiation of eosinophils (67). In addition to inducing eosinophilopoiesis, TSLP also facilitates the migration of eosinophil progenitors by promoting their ability to produce many chemokines such as CCL1, CCL22 and CXCL8 (16, 68).

Furthermore, TSLP affects the functions of basophils and mast cells (21). Indeed, TSLP stimulates the differentiation of basophils and their release of histamine and proinflammatory cytokines, as well as enhances the expression of CD203c, a specific marker of basophil activation (16, 69). In atopic asthmatic subjects, TSLP increases alarmin sensitivity of basophils by up-regulating their expression of ST2 and IL-17RB receptors, which are specifically activated by IL-33 and IL-25, respectively (70). TSLP is also able to empower mast cell biosynthesis of IL-5 and IL-13 (71). Additionally, by acting in conjunction with IL-33, TSLP stimulates mast cell production of prostaglandin D_2_ (PGD_2_), a pleiotropic mediator which is significantly involved in the pathophysiology of type 2 asthma (72).

TSLP plays a key role in the intercellular crosstalk occurring between the innate and adaptive immune responses underlying type 2 asthma (Figure 1). Within this pathogenic context, TSLP acts on human myeloid dendritic cells by enhancing their expression of major histocompatibility complex class II (MHC-II) antigens and co-stimulatory molecules CD40 and CD86 (73). Dendritic cells are also stimulated by TSLP to express OX40 ligand (OX40l), a powerful polarizing signal which drives the commitment of naïve CD4^+^ T lymphocytes towards the Th2 immunophenotype (74). Upon TSLP-induced activation, in thoracic lymph nodes OX40l + dendritic cells trigger the development of T follicular helper cells (Tfh) secreting IL-4, which is essential for the differentiation of Th2 lymphocytes and IgE isotype switching (75, 76). Moreover, TSLP stimulates dendritic cells to release the chemokines CCL17 and CCL22, which bind to the CCR4 receptor present on Th2 cells, thereby directing their migration from lung-draining lymph nodes towards the airways (16, 77). Not only dendritic cells, but also other cellular elements like CD11c^+^ monocytes/interstitial macrophages can convey TSLP-dependent stimulatory signals, which drive the maturation and expansion of Th2 lymphocytes (78). Anyway, in addition to its indirect actions worked out by dendritic cells and monocytes/macrophages, TSLP can also directly guide the commitment of CD4^+^ T cells towards the Th2 lineage (79). Another mechanism by which TSLP participates in the pathogenesis of allergic asthma is mediated by an impairment of the immuno-modulatory functions of lung T regulatory (Treg) lymphocytes (80). Specifically, TSLP inhibits Treg cellular production of interleukin-10 (IL-10), an anti-allergic and anti-inflammatory cytokine that suppresses the development of atopic immune responses (80). With regard to the pathobiology of T2-high asthma, a further contribution of TSLP refers to its property of inducing the differentiation of alternatively activated M2 macrophages, which are associated with allergic inflammation (81). Recent findings suggest that TSLP is constitutively present in the cytoplasm of human lung macrophages, where this alarmin can feed an autocrine loop leading to the release of vascular endothelial growth factor (VEGF) from TSLP-stimulated, alternatively activated M2 cells (82, 83).

### 2.2 T2-low asthma

Besides being implicated in the induction and persistence of type 2 asthma, TSLP appears to be also engaged in T2-low neutrophilic bronchial inflammation, which is predominantly mediated by the pro-inflammatory pathways powered by Th17 lymphocytes (84) (Figure 1). Notably, TSLP is capable of stimulating dendritic cells to release interleukin-6 (IL-6) and interleukin-23 (IL-23), which are essential for Th17 skewing (85, 86). In turn, Th17 lymphocytes elicit the release from endothelial cells of the potent neutrophil chemoattractant CXCL8 (11). Furthermore, TSLP can also evoke a combined activation and expansion of both Th2 and Th17 immunophenotypes, featured by a concomitant biosynthesis of IL-4 and IL-17A (86).

### 2.3 Airway remodeling

TSLP exerts its biologic effects not only on immune/inflammatory cells, but also on airway structural cells. In particular, TSLP induces the secretion of IL-6 and CXCL8 from airway smooth muscle cells, which are prompted by adjacent mast cells to release tumor necrosis factor-α (TNF-α), interleukin-1β (IL-1β) and TSLP itself (87–89). TSLP is also generated by airway fibroblasts in response to a TSLP-mediated autocrine loop, leading to an increased production of α smooth muscle actin, collagen, arginase 1 and transforming growth factor β1 (TGF-β1) (90, 91). Additionally, the angiogenetic events underlying airway vascular remodeling in asthma are significantly affected by TSLP. Indeed, VEGF release from human lung macrophages can be induced by TSLP, which is overexpressed in bronchial biopsies taken from severe asthmatic patients characterized by a mixed neutrophilic/eosinophilic inflammatory pattern, coupled with excessive neo-angiogenesis (82, 92). As a consequence, these findings underscore the crucial role played by TSLP within the intercellular axis connecting airway inflammatory and structural cells, which significantly contributes to bronchial remodeling in asthma.

## 3 Clinical outcomes of anti-TSLP therapy with tezepelumab

The only anti-TSLP biologic drug currently available in clinical practice is tezepelumab, a fully human monoclonal IgG2λ antibody which specifically binds to TSLP and impedes its interaction with TSLPR (21, 43, 93). Tezepelumab was initially compared to placebo in subjects with mild atopic asthma, undergoing allergen challenge (94). When receiving three monthly intravenous administrations of tezepelumab (700 mg), with respect to placebo control these patients experienced a relevant protection against allergen-induced bronchoconstriction, ranging from 34.0% to 45.9% attenuation of decrease in forced expiratory volume in one second (FEV_1_) at 42 and 84 days of treatment, respectively (94). Moreover, tezepelumab significantly enhanced the provocative concentration of methacholine eliciting a 20% FEV_1_ reduction (PC_20_), as well as lowered the levels of both blood/sputum eosinophils and fractional exhaled nitric oxide (FeNO) (94).

The phase 2b randomized, placebo-controlled, double-blind, multicentre PATHWAY trial was then carried out from December 2013 to March 2017 at 108 sites disseminated around 12 countries (25). All participants in this study were current non-smokers (age range: 18–75 years), suffering from uncontrolled asthma even if under treatment with an inhaled corticosteroid (ICS) consisting of fluticasone propionate (250–500 μg/day or more) or other ICS at equivalent doses, plus a combined long-acting β_2_-adrenergic agonist (LABA). Asthma was considered to be uncontrolled when at screening the ACQ-6 (6-item Asthma Control Questionnaire) score was at least 1.5. Furthermore, during the 12 months before enrolment at least two asthma exacerbations were reported, or alternatively a single severe exacerbation requiring hospitalization had occurred. Baseline pulmonary function was featured by reversible bronchial obstruction, consisting of pre-bronchodilator FEV_1_ measures ranging from 40% to 80% of predicted values, which increased by at least 12% and 200 ml after a conventional pharmacodynamic bronchodilator test (inhalation of 400 μg of salbutamol). 584 selected participants were randomly assigned either to the placebo group (148 subjects) or to one of three other arms including treatments every 4 weeks with tezepelumab administered through the subcutaneous route at doses of 70 mg (145 people), 210 mg (145 patients) and 280 mg (146 participants), respectively.

The primary endpoint of the PATHWAY study referred to the effects of 52 weeks of biologic therapy with tezepelumab on the annualized asthma exacerbation rate (AAER). In comparison to placebo, tezepelumab delayed the occurrence of the next exacerbation, and significantly (*p* < 0.001) reduced AAER by 61%, 71%, and 66% at low, intermediate and high dosages, respectively (25). Such a noteworthy therapeutic outcome did not significantly differ among patients with various blood eosinophil counts. Moreover, a *post hoc* analysis of PATHWAY verified that treatment with 210 mg of tezepelumab lowered AAER in severe asthmatic patients regardless of the eventual coexistence of nasal polyposis (95). Another *post hoc* analysis also documented that during the PATHWAY trial, tezepelumab reduced asthma exacerbations throughout all four seasons of the year (96). In regard to the secondary outcomes of PATHWAY, after 52 weeks of add-on therapy with tezepelumab, a significant ACQ-6 score improvement was detected across all three different dosage subgroups (25). Additionally, after 52 weeks tezepelumab enhanced pre-bronchodilator FEV_1_ by 150 ml, 110 ml and 120 ml when used at dosages of 280 mg, 210 mg and 70 mg, respectively (25). Tezepelumab was also able to lower the levels of several biomarkers of type 2 inflammation, including serum IgE, blood eosinophils, FeNO, periostin, IL-5, IL-13, and thymus and activation-regulated chemokine (TARC) (25, 97).

Treatment with tezepelumab was safe and well tolerated, as shown by the similar numbers of adverse reactions induced by either placebo or active therapy, mainly represented by headache, nasopharyngitis and bronchitis (25). No anaphylactic reactions were reported. Anti-tezepelumab antibodies were detected in 8.8% of recipients of placebo, as well as in 0.7%, 4.9% and 2.1% of patients treated with low, medium and high drug dosages, respectively. No neutralizing antibodies were found.

The phase 3 multicentre, double-blind, placebo-controlled and randomized NAVIGATOR study enrolled 1,061 severe asthmatic patients (age: 12–80 years) with frequent asthma exacerbations (27). 532 participants underwent a randomized assignment to placebo, whereas 529 subjects received subcutaneous administrations of tezepelumab (210 mg) every 4 weeks for 52 weeks. Patients with either more or less than 300 eosinophils/μL blood were recruited. Referring to the primary endpoint, tezepelumab significantly decreased AAER, independently of blood eosinophil counts (27). Secondary outcomes regarded symptom control, lung function, and health-related quality of life. In comparison to placebo tezepelumab improved ACQ-6, ASD (Asthma Symptom Diary) and AQLQ (Asthma Quality of Life Questionnaire) scores, as well as significantly incremented pre-bronchodilator FEV_1_ (27). Furthermore, tezepelumab lowered serum IgE levels, FeNO and blood eosinophil numbers. The most frequent adverse reactions similarly experienced by both placebo recipients and tezepelumab patients were upper respiratory tract infections, nasopharyngitis and headache. Local cutaneous reactions on injection sites occurred in 3.6% of subjects undergoing add-on therapy with tezepelumab, and in 2.6% of patients receiving placebo, respectively (27). Anti-tezepelumab antibodies were found in 4.9% of subjects treated with tezepelumab and 8.3% of placebo recipients, respectively. Neutralizing antibodies were detected in one patient belonging to the placebo group, as well in one subject assigned to treatment with tezepelumab (27).

SOURCE was another phase 3 randomized, 48-week, double-blind and placebo-controlled trial, which recruited 150 severe asthmatic patients, treated with medium-to-high doses of ICS/LABA combinations, associated with a further chronic OCS treatment (98). The main goal of this study was to ascertain the presumptive OCS-sparing action of tezepelumab, injected subcutaneously at the dosage of 210 mg every 4 weeks (98). The relevance of such a primary endpoint depends on the frequent OCS use occurring among people with severe asthma, who are thus subjected to the possible development of the well-known unwanted side effects of OCS, including glaucoma, cataract, hypertension, diabetes, adrenal insufficiency, hypertension, gastrointestinal diseases, infections, psychiatric disorders, osteoporosis, bone fracture, and reduced growth in children and adolescents (99). According to SOURCE, tezepelumab was not capable of significantly decreasing the daily OCS consumption (100). Nevertheless, tezepelumab reduced OCS intake in corticosteroid-dependent asthmatic patients with relatively high blood eosinophil counts (100). Moreover, the open-label WAYFINDER study [ClinicalTrials.gov identifier: NCT05274815] is currently performing a more comprehensive assessment of the putative OCS sparing effect of tezepelumab.

DESTINATION was a phase 3 double-blind, randomized, placebo-controlled, and long-term extension trial, which protracted over two years the evaluation of the therapeutic effects of tezepelumab (28). This study provided a further convincing evidence in favour of the sustained safety of tezepelumab, associated with its persistent efficacy resulting in a clinically meaningful AAER decrease (28). In addition, a further analysis of the results of both NAVIGATOR and DESTINATION trials showed that tezepelumab was able to induce on-treatment clinical remission of severe asthma, consisting of zeroing of disease exacerbations and OCS intake, unitedly with improvement of symptom control and lung function (101). In particular, asthma remission elicited by tezepelumab can be more easily achieved by patients with high levels of type 2 inflammatory biomarkers including FeNO and blood eosinophils, associated with a lower disease burden characterized by not frequent exacerbations, low OCS use, and a quite preserved pulmonary function (102).

CASCADE was a phase 2 double-blind, randomized, placebo-controlled trial, which enrolled patients with moderate-to-severe asthma, receiving subcutaneous injections of tezepelumab at the dosage of 210 mg every 4 weeks for 28 weeks (103). Recruitment occurred independently of baseline blood eosinophil counts. The primary endpoint of CASCADE was to evaluate the eventual anti-inflammatory effects of tezepelumab. With this aim, bronchoscopic biopsies were performed in order to analyze the potential changes induced by tezepelumab with regard to airway infiltration by inflammatory cells (103). Furthermore, the thickness of reticular basement membrane of bronchial epithelium was measured before and after therapy with tezepelumab. This biologic drug significantly reduced eosinophil accumulation within the airway submucosal layer, whereas tezepelumab did not modify the numbers of other immune/inflammatory cells including T lymphocytes, mast cells and neutrophils (104). Moreover, tezepelumab did not change the thickness of reticular basement membrane, but improved the airway hyperresponsiveness to mannitol (104), an indirect bronhoconstrictor agent which promotes mast cell degranulation. This finding about the protective effect of tezepelumab on mannitol-induced bronchoconstriction was also confirmed by the results of the phase 2 UPSTREAM trial (105). Such observations suggest that the anti-eosinophilic effects of tezepelumab contribute to explain the partial inhibition induced by this monoclonal antibody on the inflammatory component of airway hyperresponsiveness. However, the incomplete efficacy of tezepelumab could be due to the unsuccessful action of this biologic drug on airway structural remodeling.

The initial data provided by real-life studies confirm the efficacy of tezepelumab in decreasing asthma exacerbations and OCS dependence (29, 30). Additionally, tezepelumab bettered symptom control, quality of life and lung function in severe asthmatic patients featured by either T2-high or T2-low inflammatory traits, who also experienced a dramatic reduction of their need for emergency visits (29–35). According to our recent real-world findings, tezepelumab rapidly diminished the serum levels of interleukin-2 (IL-2) and VEGF in patients with either T2-high or T2-low severe asthma (106). These results are quite interesting because can contribute to deepen the knowledge about the potential ability of tezepelumab to inhibit T lymphocyte activation and angiogenesis. Indeed, as shown by the high IL-2 levels detectable in severe asthmatic patients, this cytokine is involved in the pathophysiology of both T2-low and T2-high severe asthma by promoting the differentiation of Th1 and Th2 cells, as well as by inducing airway remodeling through up-regulation of type-1 collagen expression (107–109). Furthermore, tezepelumab could suppress TSLP-dependent macrophage release of VEGF induced by T2-high (IL-4, IL-13) and T2-low (lipopolysaccharide) proinflammatory stimuli (82, 83).

Because asthma and its frequent comorbidity nasal polyposis (NP) share common pathogenic mechanisms, it is noteworthy that tezepelumab has been recently shown to be therapeutically effective also in NP treatment (110, 111). Indeed, the WAYPOINT trial demonstrated that tezepelumab treatment decreased the size of nasal polyps and the severity of sinonasal symptoms and nasal congestion, as well as the needs for OCS therapy and surgical polypectomy (111).

Taken together, the above studies suggest that tezepelumab may be very effective in a wide subpopulation of patients with severe asthma, triggered by either multiple factors involved in T2-high disease, as well as by pathogenic mechanisms implicated in T2-low airway inflammation. However, the main limitation regarding the use of tezepelumab appears to be the lack of specific biomarkers capable of guiding the choice of asthma physicians towards this monoclonal antibody.

## 4 Conclusions

Several asthma triggers including aeroallergens, respiratory pathogens and airborne pollutants damage the airway epithelium and activate innate immune receptors expressed by bronchial epithelial cells, thus stimulating them to release alarmin cytokines. Among the latter, TSLP plays a key pathogenic role in the induction, persistence and amplification of both T2-high and T2-low asthma. Therefore, because of its upstream strategic position, TSLP represents a suitable molecular target for add-on treatments aimed to disrupt epithelial-driven proinflammatory pathways leading to the development of severe asthma. In fact, both randomized clinical trials and recent real-world studies emphasize the therapeutic utility of the anti-TSLP monoclonal antibody tezepelumab. This biologic drug is indeed very effective in decreasing asthma exacerbations and OCS intake, as well as in ameliorating symptom control and lung function. Hence, tezepelumab makes it possible for many patients with either type 2 or non-type 2 severe asthma to achieve the realistic goal of clinical remission, which translates into a very relevant improvement of their quality of life.
